# Barriers and enablers to utilisation of the WHO surgical safety checklist at the university teaching hospital in Lusaka, Zambia: a qualitative study

**DOI:** 10.1186/s12913-022-08257-y

**Published:** 2022-07-09

**Authors:** Judith Munthali, Chiara Pittalis, Leon Bijlmakers, John Kachimba, Mweene Cheelo, Ruairi Brugha, Jakub Gajewski

**Affiliations:** 1 University Teaching Hospital, Nationalist Rd, Lusaka, Zambia; 2 Institute of Global Surgery, Royal College of Surgeons in Ireland, Dublin, Ireland; 3 Department for Health Evidence, Radboud University Medical Centre, Nijmegen, The Netherlands; 4 Department of Surgery, Surgical Society of Zambia, University of Zambia University Teaching Hospital, Lusaka, Zambia

**Keywords:** Surgical safety checklist, Adverse events, Patient safety, Zambia

## Abstract

**Background:**

Surgical perioperative deaths and major complications are important contributors to preventable morbidity, globally and in sub-Saharan Africa. The surgical safety checklist (SSC) was developed by WHO to reduce surgical deaths and complications, by utilising a team approach and a series of steps to ensure the safe transit of a patient through the surgical operation. This study explored barriers and enablers to the utilisation of the Checklist at the University Teaching Hospital (UTH) in Lusaka, Zambia.

**Methods:**

A qualitative case study was conducted involving members of surgical teams (doctors, anaesthesia providers, nurses and support staff) from the UTH surgical departments. Purposive sampling was used and 16 in-depth interviews were conducted between December 2018 and March 2019. Data were transcribed, organised and analysed using thematic analysis.

**Results:**

Analysis revealed variability in implementation of the SSC by surgical teams, which stemmed from lack of senior surgeon ownership of the initiative, when the SSC was introduced at UTH 5 years earlier. Low utilisation was also linked to factors such as: negative attitudes towards it, the hierarchical structure of surgical teams, lack of support for the SSC among senior surgeons and poor teamwork. Further determinants included: lack of training opportunities, lack of leadership and erratic availability of resources. Interviewees proposed the following strategies for improving SSC utilisation: periodic training, refresher courses, monitoring of use, local adaptation, mobilising the support of senior surgeons and improvement in functionality of the surgical teams.

**Conclusion:**

The SSC has the potential to benefit patients; however, its utilisation at the UTH has been patchy, at best. Its full benefits will only be achieved if senior surgeons are committed and managers allocate resources to its implementation. The study points more broadly to the factors that influence or obstruct the introduction and effective implementation of new quality of care initiatives.

**Supplementary Information:**

The online version contains supplementary material available at 10.1186/s12913-022-08257-y.

## Background

There is evidence that a lack of safety protocols in surgery could lead to a range of surgical adverse events [1], contributing to preventable deaths [2]. Avoidable complications during surgery commonly arise because of factors such as: operating on the wrong patient; using the wrong procedure or at the wrong site; inadequate anaesthesia and surgical skills and equipment; lack of readiness to manage unanticipated blood loss; and non-sterile equipment and surgical items and sponges left inside body cavities of patients, resulting in sepsis; and failure in non-technical skills such as communication and teamwork [2]. Patient safety and measures to ensure optimal outcomes of surgery are particularly important in Africa, where patients are twice as likely to die after surgery compared to the global average, and the risk of death following perioperative complications is significantly greater than in other regions [3].

In 2008 the World Health Organization (WHO) developed the Surgical Safety Checklist (SSC) to improve surgical patient safety. The SSC reinforces safety practices in surgery and fosters better communication and teamwork between clinical disciplines [4]. It is designed to allow the surgical team (surgical providers, anaesthesia providers, nurses and others) to discuss, agree and check important details about each surgical case at three key time-points in the normal flow of a surgical procedure, namely: i) briefing phase before induction of anaesthesia, ii) “time out” period after induction and before surgical incision and iii) debriefing phase after wound closure, before leaving the operating room [5]. A substantial body of evidence has demonstrated considerable benefits in the use of the SSC to improve safety [6], reduce complication rates [6, 7] and mortality rates; and ensure critical incident reporting [8].

In Zambia, data on rates of perioperative mortality are not systematically reported. However, a study conducted at the University Teaching Hospital (UTH) estimated that over 60% of perioperative deaths registered in 2012 at the hospital were avoidable or potentially avoidable [9]. Key factors identified as contributing to avoidable mortality at UTH included, among others, undue delays in surgery, inadequate preparation of the patient, and poor perioperative and post-operative care (both surgical and in anaesthesia) [9]. The study also compared findings with historical data from 1987 and found no improvements in perioperative mortality, concluding that many deaths remained avoidable [10]. This suggested the urgent need to enhance quality and safety of surgical service delivery at UTH and countrywide.

The WHO SSC was first introduced at UTH around 2009/2010 as an initiative by the College of Surgeons of East, Central and Southern Africa (COSECSA) in collaboration with surgeons from UTH. Additionally in 2015, the UK Royal College of Nursing (RCN), in partnership with the Zambia Union of Nurses Organization (ZUNO) and the Zambia Operating Theatre Nurses Interest Group (ZOTNIG), conducted formal training in the utilisation of the SSC in an attempt to improve surgical safety. The National Surgical, Obstetric and Anaesthesia Plan (NSOAP) 2017–2020 included an explicit commitment to protecting surgical patients from avoidable complications and improving health outcomes and endorsed the use of the WHO SSC.

This study aimed to explore barriers and enablers to the utilisation of the WHO SSC at UTH to identify any shortcomings and potential areas for improvement.

## Methods

The study employed an exploratory qualitative approach involving semi-structured interviews with key members of the surgical teams at the operating theatre departments of UTH. Located in the capital city Lusaka, UTH is the largest hospital in Zambia with 9960-recorded surgical operations in 2018. The study is reported in accordance with the Consolidated Criteria for Reporting Qualitative Research (COREQ) [11] (details in Appendix).

### Study sample and data collection

Sixteen members of the surgical teams at UTH participated in the study. They were selected using purposive sampling, with the following inclusion criteria: medical doctors, anaesthesia providers, nurses and theatre support staff (i.e. theatre porters and maids oriented to work in the operating rooms), who were working full time at UTH theatre departments and were willing be interviewed. Four participants from each cadre were included in the study. The nurses included only those with a specialty training in perioperative care and not the general nurses who worked in the operating rooms intermittently as part of their rotation across departments. An interview guide was developed based on a review of relevant published studies and was piloted prior to administration. The open-ended questions explored the views and experiences of the surgical team members on the utilisation of SSC and factors related to the implementation of the checklist.

Data were collected between December 2018 and March 2019 by the first author, who is a female, operating theatre nurse by training, with a Master’s in Public Health and familiar with qualitative research methods. Participants were contacted in advance via phone to explain the purpose of the study and schedule a suitable time for the face-to-face interviews, which were conducted individually in a quiet space at UTH to ensure the privacy and anonymity of respondents. Participant information was read out aloud and questions were answered before each interviewee signed the consent form. Interviews lasted 30 to 60 minutes and were audio recorded. They were then transcribed verbatim by the first author. To ensure anonymity and confidentiality, a coding system was used delinking interviews from participant characteristics. There was no financial incentive for taking part in this study.

### Data analysis

**Fig. 1:**
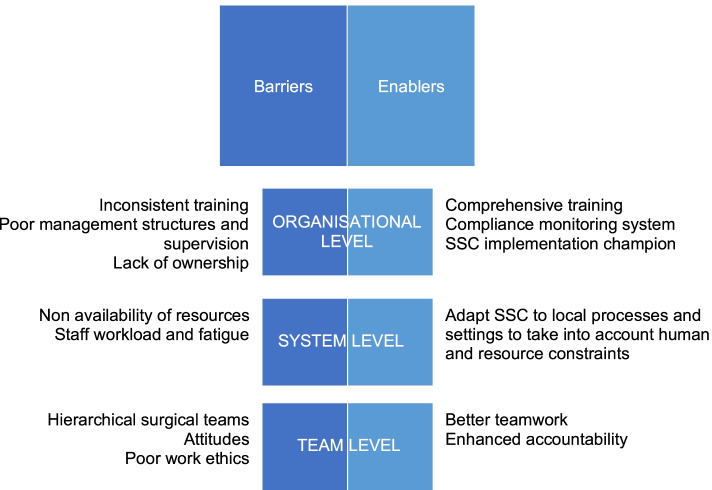
Factors related to SSC utilisation at UTH - emerging themes

Anonymised transcripts were analysed manually and the process was recorded in MS Word. Thematic analysis was performed following the steps proposed by Braun and Clarke [12]. The first author started by reading the transcribed interview data to identify any factors mentioned by respondents that may influence the utilisation of the SSC. After this first familiarisation with the data, all factors affecting SSC utilisation were coded accordingly. Subsequently, conceptually related factors were combined under themes (see Fig. 1). These were discussed and reviewed together with the other authors before the final coding tree was agreed upon.

The coding followed a top-down approach, informed by some elements of the model proposed by Russ et al. 2015 [13] which provided guidance on common barriers and facilitators surrounding implementation of the WHO SSC in hospital settings. However, the Russ et al. model was developed in high-income settings, so through an iterative process the authors continuously revised and adapted the codes and themes until they were satisfied they accurately reflected the data and the realities in Zambia.

Finally, emerging themes were grouped into three broad overarching categories (at organisational, system and team levels), following the model by Russ et al. [13], which was adapted to the purpose of this study. Organisational themes relate to organisational structures, policies and standards, including in relation to staff training and supervision; and organisational safety culture and priorities. System level themes relate to the integration (or lack of) of the checklist into existing systems and procedures. At the team level themes relate to teamwork (e.g. communication, cohesion), team structure (e.g. leadership and power dynamics), and team buy-in of the checklist.

## Results

Most interview respondents indicated that the WHO SSC was not used consistently at UTH, despite the MoH endorsement of the SSC as standard practice.

### Barriers to SSC utilisation

Respondents reported a range of factors hindering consistent utilisation of the SSC before each surgery, as follows.

#### Organisational barriers

##### Surgical team - inconsistent training

Study participants reported that, after the initial training, there had not been any further training opportunities for staff rotating from other departments or newly hired. As a result, the checklist was not followed in the operating rooms run by new or rotating staff (mainly emergency OTs), who had a poor understanding of the SSC intended purpose and benefits. In these OTs, senior medical and nursing staff who had undergone training rarely shared their learning or encouraged the use of the SSC among their team members with no training.

##### Lack of ownership and management structures, including supervision

Interviewees stated that poor ownership of the SSC initiative, among other reasons, was because some of the senior staff did not attend the initial SSC training. Consequently, these staff members failed to fully grasp the value of the SSC and perceived it as an unnecessary imposition.

(MD6) *“Yes, very senior staff, especially some medical doctors who have been operating for years without any recorded AEs/complications, may think that the tool is being imposed on them by the westerners, and feel it is not important and won’t utilise the tool. If they are trained and shown actual data from other countries they may change and they would do well to be part of the adverse events audit.”*

Study participants also indicated that there was no supervision nor any formal oversight measure in place, such as a designated person in-charge, to ensure consistent use of the SSC at UTH operating theatre departments. This resulted in lack of accountability in instances of non-adherence.

(AP1) “*Where I work from I rarely see among other leaders, consultants to check on how the junior medical doctors are working. In short, there is no leader figure ensuring that the SSC is utilised consistently at UTH, despite the fact that there are a lot of new staff who haven’t yet been trained on the application of the SSC, and who find it difficult to fit in.”*

#### System barriers

##### Non-availability of resources

According to study participants, the often-occurring non-availability of essential surgical equipment and supplies at the time of an operation had an indirect, negative impact on the use of the SSC. All participants consistently indicated that in these instances management of the OTs was challenging and a considerable amount of time was spent on searching for and borrowing resources from other theatre rooms, or sending faulty equipment for repairs.

This delayed the operations and took away some of the actual surgery time, which could only start when all required resources were present in the operating room, with a knock-on effect on the surgical list. As a result, in these instances the surgical teams tried to make up for the lost time by skipping the SSC.

(MD6) *“You would want to be out of theatre as soon as possible...and therefore, you end up not utilising the SSC. This is because the challenges with the availability of material resources are huge and come in the form of less numbers or nothing at all to use for most times, such as equipment, instruments, consumables and there are no comfortable well-ventilated theatres”.*

##### Staff workload and fatigue

Additionally, there was a reported widespread shortfall of essential surgical staff such as nurses, anaesthesia providers and support staff to cover all operating rooms. Participants reported that non-specialised nurses (non-perioperative) were often allocated to work in the surgical departments to fill these gaps. They were usually given on-the-job orientation to be able to work in the operating theatres, but the use of the SSC was not routinely covered.

(PN9) *...staffing is also a huge barrier … . [there are] very few nurses [available] against all the operating rooms that are open, the recovery room and theatre sterile supply unit to prepare surgical sets continuously. You end up having the medical doctors conducting certain operations alone, while the nurses are scrubbed up in the other operating rooms and you find that the SSC will not be used.”*

Inadequate human resources were also reported to contribute to high workload and fatigue of surgical teams, particularly the ones handling emergency operating rooms. Respondents stated that these staff shortages, coupled with lack of protocols to enforce SSC utilisation before every surgical operation, meant that when clinicians were overwhelmed, they skipped the SSC. This was also common in instances where the SSC printed out copies were misplaced. As reported, this in turn resulted in failure to conduct adequate handovers of cases at the end of shifts, which occasionally led to recording incomplete medical details about patients undergoing surgery.

(PN9) *“...you have the surgical ‘firm’ of medical doctors ‘On Call’ and other firms rushing in their emergency cases and adding to the already prevailing high workload and also the nature of the urgency of doing these cases. If not reminded, due to the workload the medical doctors and support staff can actually work without using the SSC”.*

#### Team barriers

The absence of standard operating procedures to guide the work of the surgical teams and an assigned champion to ensure implementation of the SSC resulted in its inconsistent application. Rather than following a team approach, participants reported excessive influence of individuals whose behaviour and attitude could either drive or hinder the use of the checklist. The decision whether to implement the checklist or not was linked to individual team members’ attitude towards it, and more broadly, with their professional attitude and work ethics. This was due to several reasons as described below:

##### Hierarchical surgical team structure

Participants reported that the structure of surgical teams at UTH was highly hierarchical. Within this structure, the surgeon was regarded as having a ‘higher’ central, decision-making role, while the other team members (anaesthesia providers and nurses) were perceived to be in a ‘lower’ subordinate position. These power dynamics within the team meant that the attitude of team members at the top of this hierarchy towards the checklist usually prevailed over others and guided the manner in which decisions to use the checklist were made. Respondents in ‘lower’ positions in the team indicated that often it was they who suggested following the SSC, but in some instances, the surgeon leading the operation did not support its use.

(TSS13) *“Sometimes you find that you need to remind the medical doctors as junior staff to utilise the SSC, like ‘let’s do this … let’s do this’. However, they would not want to apply the SSC and you then just start conducting the surgery. Eventually, as a junior staff, you mostly follow what senior staff take on board (...) with regard to keenness towards utilisation of the SSC.*”

The hierarchical and surgeon-centred structure of the surgical team played also a role in the rollout of the initial SSC training offered to UTH surgical staff in 2015. According to an interviewee, some surgeons (these being perceived as in a ‘senior or higher’ position) chose not to attend as they did not want to receive training from a person whom they considered junior to them (at the time the SSC training was delivered by a nurse).

(MD8) *“It is an issue of specific team members not wanting to attend training that brings and mixes the entire surgical team in one room.*”

##### Attitudes

Negative attitudes towards the SSC by some members of the surgical team also affected its utilisation. Participants reported that some surgeons often ‘*rushed*’ to proceed with a case, seeing the SSC as an unnecessary *‘delay’*. In some instances, such behaviour and lack of support from senior or ‘perceived higher-level’ team members made the staff in junior or perceived ‘lower’ positions reluctant to further suggest using the SSC for subsequent cases.

The interviewed senior surgeons who were supportive of the use of the SSC confirmed the presence of such behaviour among some of their peers. They acknowledged that such dismissive attitude had negative consequences for the team dynamics, and ultimately led to lack of a team approach to and poor utilisation of the SSC.

(MD6*) ‘I think mostly it is a misconception by seniors that surgery revolves around them and not the other members of the surgical team. It is important to respect the opinions of each member of the team and discourage intimidation of junior team members for them to feel part of the team.’*

Another reported issue was the occasional occurrence of ‘*intimidating’* behaviours, where some junior or perceived ‘lower position’ staff (such as anaesthetists) were told that they ‘*want to waste time*’ after they had suggested utilisation of the SSC prior to the operation.

(AP1) “*The only problem that I have mentioned even before, is that the other team members think anaesthesia providers delay when signing in the patient and would then force the use of the SSC”.*

This in turn further deteriorated the already poor team dynamics and reinforced the hierarchical structure. The effect of this reported ‘*intimidation*’ behaviour was illustrated in the following way:

(TSS14) *“Like to me, personally, I do try to engage seniors when they do something I perceive is not in line with [good] practice such as utilisation of the SSC and their responses vary as individuals. I think that is why we have even forgotten a lot about the SSC use, because this affects our confidence levels.”*

##### Poor work ethics

Participants also reported that the utilisation of the SSC was undermined by the poor work ethics of some of the team members. One of the issues identified by respondents was the late reporting for work, which led to failure to form surgical teams on time and delayed the start of the operation. To compensate for the time lost, the surgical teams proceeded with surgery without applying the SSC. This had a knock-on effect on other surgical teams operating later on in the day. A delay caused by one team could derail the whole operating room schedule, forcing the next team to work under unnecessary time pressure.

(MD5) *“Some of the barriers regarding non-utilisation of the SSC are that we are supposed to start surgery at 08.00 hours but because of the late coming by some team members we normally start work at about 09.30 - 10.00 hours... then they would want to catch up with the lost time and as such omit SSC use.”*

### Enablers to SSC utilisation

#### Organisational enablers

Identified enablers corresponded to the barriers presented above.

##### Training

Firstly, training on SSC for all new surgical staff members was deemed necessary before rotation to the theatre rooms from other departments. Refresher training for all existing staff was also suggested as a way to improve adherence to the checklist.

(MD7) *“It is better to keep training all new staff on SSC utilisation and I think there is a need to plan on our calendars that we need to regularly hold workshops to update our knowledge. The preoperative phase is equally very important and there is a need to also empower surgical ward nurses with skills on pre-operative preparation of patients and include them in lessons about safe surgery.”*

##### Monitoring system and designated champions

The respondents suggested that better enforcement of the formal system already put in place by the Ministry of Health, termed Service Quality Assessment (SQA), could improve SSC utilisation and accountability in the operating rooms at UTH. This involved the use of a tool to monitor compliance. However, respondents stated that this needed to be reinforced with the allocation of specific funds to ensure its proper application. It was also noted that implementation champions would be needed to ensure consistency in the use of the SSC. The champions would ensure that compliance with SSC use is measured and documented, would provide relevant feedback to the theatre teams and facilitate communication with hospital management. Additionally, setting up teams to periodically evaluate the SSC utilisation given monitoring patient safety was also seen as an essential component of standard surgical practice.

(PN9) “*Another way to enhance SSC utilisation would be for management to bring on board funded initiatives to strengthen monitoring of the SSC utilisation, because it will then make the staff know that they have to be answerable to someone.”*

#### System enablers

##### Adaptation of SSC to the local setting

There was a reported need to adapt the SSC to the UTH’s local setting and systems to enhance application in the operating rooms. Participants suggested that a review of how the SSC was implemented would help to adapt it better to the local setting, considering the handling of emergencies and the deficiencies in the human and material resources.

Additionally, respondents reported the need to ensure more visibility of the SSC tool by displaying it in the OT room in a clear place, such as on a wall for example. They stated this would act as a reminder for the entire team, especially the new staff, to follow the steps during the execution time in the operating rooms.

#### Team enablers

##### Teamwork and enhanced accountability

Participants made several suggestions for improving the overall management of the surgical theatre in order to overcome challenges related to negative attitudes and poor work ethics and lack of accountability. These included improvements in surgical team functionality:

(AP2) *“What we need first and foremost is agreeing on the start time for procedures. Basically the whole team should start together instead of what is happening currently, where at times some team members just come into theatre along the way, meaning their input won’t be known and they miss the briefing and ‘sign in’ of the SSC process.*

As well as in the division of roles and responsibilities in the workplace:

(AP2) *“Senior staff also need to delegate leadership roles to juniors for them to learn the skills ( … ), instead of what happens where sometimes the seniors usually take up a lot and fail to do important practices like SSC utilisation. Now one person does everything alone then safety is compromised.”*

## Discussion

The main objective of this study was to assess barriers and enablers to utilisation of the WHO SSC at UTH theatre departments. The findings demonstrated inconsistent use of the SSC, linked to a range of organisational, system and team level factors. While the findings pertain to the SSC, they point to broader deficiencies in the management of the surgical theatres, tensions between hierarchical and team approaches, and a lack of commitment and involvement on the part of some senior surgeons in the introduction and implementation of new quality improvement initiatives.

The organisational barriers firstly related to lack of adequate training offered to all surgically active staff at UTH. After the initial training, there was no follow up to ensure consistent implementation and compliance. The introduction of a checklist does not automatically lead to improved outcomes, but extensive education and sustainable training regarding its use are required to improve buy-in among surgical staff [13] and deal with an influx of new personnel at the operating theatres.

Although the NSOAP indicated some high-level commitment toward improving patient outcomes with the SSC, there was no ongoing role or involvement of the Ministry of Health (MoH) in its implementation. Lack of a high-level directive and commitment of senior staff, identified in this study, contributed to poor local ownership and lack of champions who could then drive and sustain the implementation of the SSC across different operating rooms at UTH.

This is a common barrier to SSC utilisation reported by others across different settings [14], yet it has been demonstrated that the presence and role of local champions is critical in promoting improvements in SSC utilisation [2, 13, 15, 16]. Similarly, the introduction of dedicated teams to periodically monitor and evaluate outcomes was identified as an essential component of strengthening SSC execution [17]. Accountability is unlikely to be achieved without institutional support and leadership within the surgical teams [18].

Respondents also indicated that in order to improve utilisation rates there was a need for more attention to the way the SSC tool was applied in the particular setting and systems at their hospital, and how this process could be adapted to take into account logistical and resource challenges faced by local teams. For example, our study found that the SSC protocols were not displayed in some operating rooms at UTH, which was reported as an implementation barrier. Relatively simple and inexpensive changes such as wall-mounted checklists could enhance team engagement and encourage SSC utilisation [19, 20]. There is evidence that adapting the SSC to local settings promotes the feeling of ownership and increases SSC compliance in the operating rooms [13, 21–25].

Systemic inefficiencies in the surgical departments at UTH related to staff, equipment and supply shortages were also identified as barriers to SSC utilisation by our study participants. Consequently, surgical staff felt fatigued and unmotivated to fulfil the demands of the SSC utilisation. Fatigue combined with lack of standard patient safety protocols create a negative feedback loop making surgical staff prone to making mistakes [26, 27].

Other studies highlighted that improvements in hospital practice and resources increased the use of the SSC [21, 28–30]. However, findings from studies in Cambodia and Moldova [29, 31] reported that material resources were not the primary barrier to checklist implementation in these settings, and a good level of SSC implementation was achieved despite limitations in resources. This suggests potential contextual differences across geographical regions. This should be explored in further studies.

In our study surgical team dynamics, with the surgeon at the centre in a power-holding position, were a critical factor to sustained implementation of the SSC: when the lead surgeon had a negative attitude towards the SSC this acted as a clear barrier to its use among the rest of the team. The influence of power dynamics and hierarchies on relations and functionality of surgical teams is well-known phenomenon in the surgical workforce literature [32, 33], and can have an effect on compliance with safety protocols such as the SSC [13, 26, 28, 34].

A 2013 study across 10 African countries further confirmed that organisational and cultural barriers can be critical to long-term utilisation of the SSC, and strong supportive leadership is needed to overcome them [35]. Two particular factors identified in our study which need to be addressed through a cultural change are poor work ethics and poor team approach to surgery. Further research would be beneficial to explore in more detail the types of (and reasons for) staff routinely arriving late for surgery, and what reasons are behind senior surgeons skipping the SSC due to lack of time, as reported in our study. The literature suggests that engaging in dual public-private practice, common among many clinicians, may add pressure on public sector work duties [36], so it may be interesting to assess whether this plays a role in the late presentation for work observed in our study.

The WHO SSC is an important tool in the operating room environment [37–39]. Its introduction is a behavioural intervention, which is complex and challenging. Achieving successful implementation and good adherence rates, requires time and a change in the safety culture among clinicians [40]. Although training programmes are essential to kick start the culture of using the checklist in facilities where it has not been used before, as demonstrated by the Zambian experience they are likely to achieve little long-term impact without a strong and sustainable system to reinforce its use after the initial intervention is finished.

### Study strengths and limitations

The strength of this study is that it is the first in-depth qualitative study in the Zambian setting exploring user-related barriers and facilitators to the utilisation of the WHO SSC. Sixteen [16] in-depth interviews were conducted with four types of surgical personnel, providing a comprehensive understanding of the setting in which the teams work and implement the SSC. The lead researcher was a senior operating theatre nurse working at UTH, familiar to all of the study participants. This was instrumental in obtaining good quality data and deeper accounts from study participants, who were likely to engage in a frank conversation with the interviewer. The importance of this paper is that, while reporting and discussing findings on the utilisation of the surgical safety checklist at a national teaching hospital in Africa, it points to a broader set of dynamics around hierarchical versus team working and a patchy involvement of senior surgeons in the embedding of quality of care initiatives.

This study has several limitations. Firstly, it was not designed to capture and quantify the extent to which the SSC is being utilised and further studies are needed to unpack measure and analyse the factors determining compliance. Secondly, the evidence presented in this publication is self-reported, which is a limitation due to potential biases; and in particular the willingness, openness and/or reluctance of participants to discuss the factors determining SSC utilisation with the lead author, who was a staff member at the UTH operating theatre. Finally, within the resources available in this study, it was not possible to conduct a survey of operating theatre staff, nor undertake a detailed, first-hand observation of the implementation of the surgical checklist. However, the use of qualitative interviews was appropriate to developing insights and elucidating the factors that led to sub-optimal utilisation of the surgical safety checklist at Zambia’s national teaching hospital.

## Supplementary Information

**Additional file 1.**

## Data Availability

The datasets generated and/or analysed during the current study are not publicly available due to confidentiality but are available from the corresponding author on reasonable request.

## Abbreviations

AE: Adverse Event
COSECSA: College of Surgeons of East, Central and Southern Africa
MoH: Ministry of Health
NHRA: National Health Research Authority
NSOAP: National Surgical, Obstetrics and Anaesthesia Plan
OT: Operating Theatre
RCN: UK Royal College of Nursing
SSC: Surgical Safety Checklist
UNZABREC: University of Zambia Biomedical Research Ethics Committee
UTH: University Teaching Hospital
WHO: World Health Organisation
ZOTNIG: Zambia Operating Theatre Nurses Interest Group
ZUNO: Zambia Union of Nurses Organization
